# Notch and LIM-homeodomain protein Arrowhead regulate each other in a feedback mechanism to play a role in wing and neuronal development in *Drosophila*

**DOI:** 10.1098/rsob.240247

**Published:** 2025-04-30

**Authors:** Jyoti Singh, Dipti Verma, Bappi Sarkar, Maimuna Sali Paul, Mousumi Mutsuddi, Ashim Mukherjee

**Affiliations:** ^1^ Department of Molecular and Human Genetics, Banaras Hindu University, Varanasi, Uttar Pradesh, India

**Keywords:** Arrowhead, Notch signalling, Wingless, Chip, *Drosophila*

## Abstract

The Notch pathway is an evolutionarily conserved signalling system that operates to influence an astonishing array of cell fate decisions in different developmental contexts. To identify novel effectors of Notch signalling, we analysed the whole transcriptome of *Drosophila* wing and eye imaginal discs in which an activated form of Notch was overexpressed. A LIM-homeodomain protein, Arrowhead (Awh), was identified as a novel candidate that plays a crucial role in Notch-mediated developmental events. *Awh* alleles show strong genetic interaction with Notch pathway components. Awh loss-of-function upregulates Notch targets Cut and Wingless. Awh gain-of-function downregulates Notch targets by reducing the expression of the ligand Delta. Consequently, the expression of the Wingless effector molecule Armadillo and its downstream targets, Senseless and Vestigial, also gets downregulated. Awh overexpression leads to ectopic expression of *engrailed*, a segment polarity gene in the anterior region of wing disc, leading to patterning defects. Additionally, Notch gain-of-function-mediated neuronal defects get significantly rescued with Awh overexpression. Activated Notch inhibits Awh activity, suggesting a regulatory loop between Awh and Notch. Additionally, the defects caused by Awh gain-of-function were remarkably rescued by Chip, a LIM interaction domain containing transcriptional co-factor. The present study highlights the novel feedback regulation between Awh and Notch.

**Figure:**
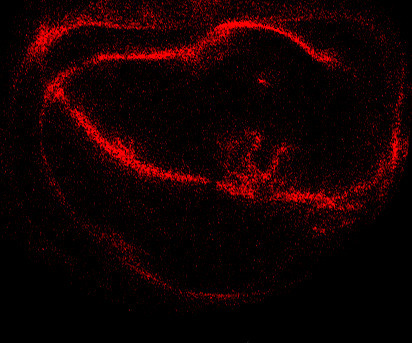

## 1. Introduction

The Notch pathway is an evolutionarily conserved signalling system, and it is highly pleiotropic in nature because it regulates a spectrum of cellular events such as cell fate determination, differentiation, proliferation, apoptosis and stem cell maintenance during development [1–4]. Notch is synthesized as a 300 kDa precursor protein that is subjected to its first cleavage during its maturation in the trans-Golgi network by furin-like convertases (S1 cleavage), resulting in a N-terminal extracellular domain of 180 kDa and a C-terminal transmembrane intracellular domain (NTM) of 120 kDa [5]. This heterodimeric Notch receptor is transported to the plasma membrane where it interacts with ligands of the DSL family (*Drosophila* Delta and Serrate (Jagged in mammals) and *C. elegans* LAG-2) expressed in the adjacent cell. This receptor–ligand binding leads to second proteolytic cleavage (S2) by a disintegrin and metalloprotease (ADAM) family of metalloproteases in the extracellular region of the NTM [6]. This is followed by an intramembranous cleavage (S3) by γ-secretase complex (Presenilin, Nicastrin, PEN-2 and APH-1) and results in the release of Notch intracellular domain (NICD) from the membrane [7,8]. Then, Importin-α3 mediates the translocation of NICD to the nucleus [9]. NICD associates with the DNA-binding protein CSL (mammalian CBF1/*Drosophila* Su(H)/ *C. elegans* Lag-1) in the nucleus and facilitates the displacement of transcriptional co-repressors. The NICD–CSL complex then recruits Mastermind and other transcriptional co-activators leading to activation of Notch target genes such as the *Enhancer of Split* (*E(spl)*) complex genes in *Drosophila* [3,10–12]. These bHLH transcription factors, in turn, repress *achaete–scute complex* (As-C) proneural genes. Depending on the cellular context, NICD–CSL activation complex can also activate other Notch target genes such as *wg, cut, string/CDC-25* and *c-myc* [10,13]. The increasingly complex regulatory mechanisms of Notch signalling account for the multitude of functions exhibited by Notch during development.

Similarly, Wingless (Wg) acts as a ligand and activates Wg signalling by binding to the receptor Frizzled. Wg signalling is an evolutionarily conserved pathway that regulates pivotal roles in cell fate determination during embryonic development. Upon binding with the receptor, it leads to dual phosphorylation of Lrp6 by GSK-3-β (Shaggy) and casein kinase-1 (CK1) in cytosol and shifting of the whole complex to plasma membrane along with Dishevelled (Dsh). This complex aids in stabilization of β-catenin (Armadillo). β-catenin moves to the nucleus and leads to activation of downstream target genes *senseless (sens), distal-less (dll) and vestigial* (*vg*). In the absence of Wg, a complex of Axin, adenomatous polyposis coli (APC), GSK3-β, CK1 and β-catenin is present in cytosol. Armadillo is phosphorylated by GSK3-β in this complex and directed to proteasomal degradation [14].

These two signalling pathways, Notch and Wg, are the major regulators of compartmentalization and fate determination of wing blade, dorsal–ventral boundary, wing hinge region as well as wing vein development of *Drosophila melanogaster* [15]. During early developmental events, *engrailed (en*), a homeodomain-containing segment polarity gene is expressed in cells adjacent to wg-expressing cells and maintains *wg* transcription. Wg is also crucial to maintaining En levels, indicating an autoregulation of its expression through paracrine feedback loop [16]. Feedback regulation is a fundamental approach that helps cells uphold proper homeostasis and govern a range of cellular processes. It is a dynamic system that provides adaptability, resilience and stability to cellular systems.

Here, we present functional characterization of a novel effector of Notch signalling identified through transcriptome analysis. At this end, whole transcriptome profiling of the *Drosophila* wing and eye imaginal discs overexpressing activated form of Notch was carried out [17]. Arrowhead (Awh), a LIM-homeodomain (LIM-HD) protein, was identified as a novel candidate, which plays a crucial role in Notch-mediated developmental events. LIM-HD proteins are characterized by the presence of two tandem LIM domains (named for the first defining members: Lin-11, Isl-1 and Mec-3) and a homeodomain [18–20]. The LIM domain consists of two unique zinc fingers that are rich in cysteine and connected by a short two-amino acid linker. It acts as a transcription factor. It is a highly conserved molecule across the taxa; its mammalian ortholog, lhx8, has been identified to have a role in neuronal development via the WNK signalling pathway [21]. Awh is well known for its role in the establishment of a proper number of precursor cells in incorporate imaginal tissues such as abdominal histoblast and salivary gland imaginal rings in *Drosophila*. Interestingly, both loss-of-function and gain-of-function alleles of Awh affect early developmental events. The ectopic expression of Awh in excorporate imaginal discs leads to elimination of cells [22].

Here we report that a LIM-HD protein Awh was identified through a transcriptome analysis from the activated Notch-induced wing and eye imaginal discs in which Awh was downregulated. Real-time PCR analysis confirmed that overexpression of activated form of Notch downregulated *Awh* transcripts. *Awh* mutant alleles displayed strong genetic interaction with *Notch* pathway components. It was seen that Awh loss-of-function upregulates Notch targets, Cut and Wg. Gain-of-function of Awh downregulates these Notch targets, Cut and Wg, and reduction of these targets was mediated through the downregulation of the ligand, Delta, without altering Notch receptor levels. Here we also show that Awh plays a major role in wing development by regulating Notch-mediated Wingless signalling. Additionally, neuronal defects caused due to Notch gain-of-function were rescued with Awh overexpression. Interestingly, activated Notch inhibits Awh activity, highlighting the novel feedback regulation between Awh and Notch.

## 2. Results

### 2.1. *Awh* genetically interacts with Notch pathway components

**Figure 1.**
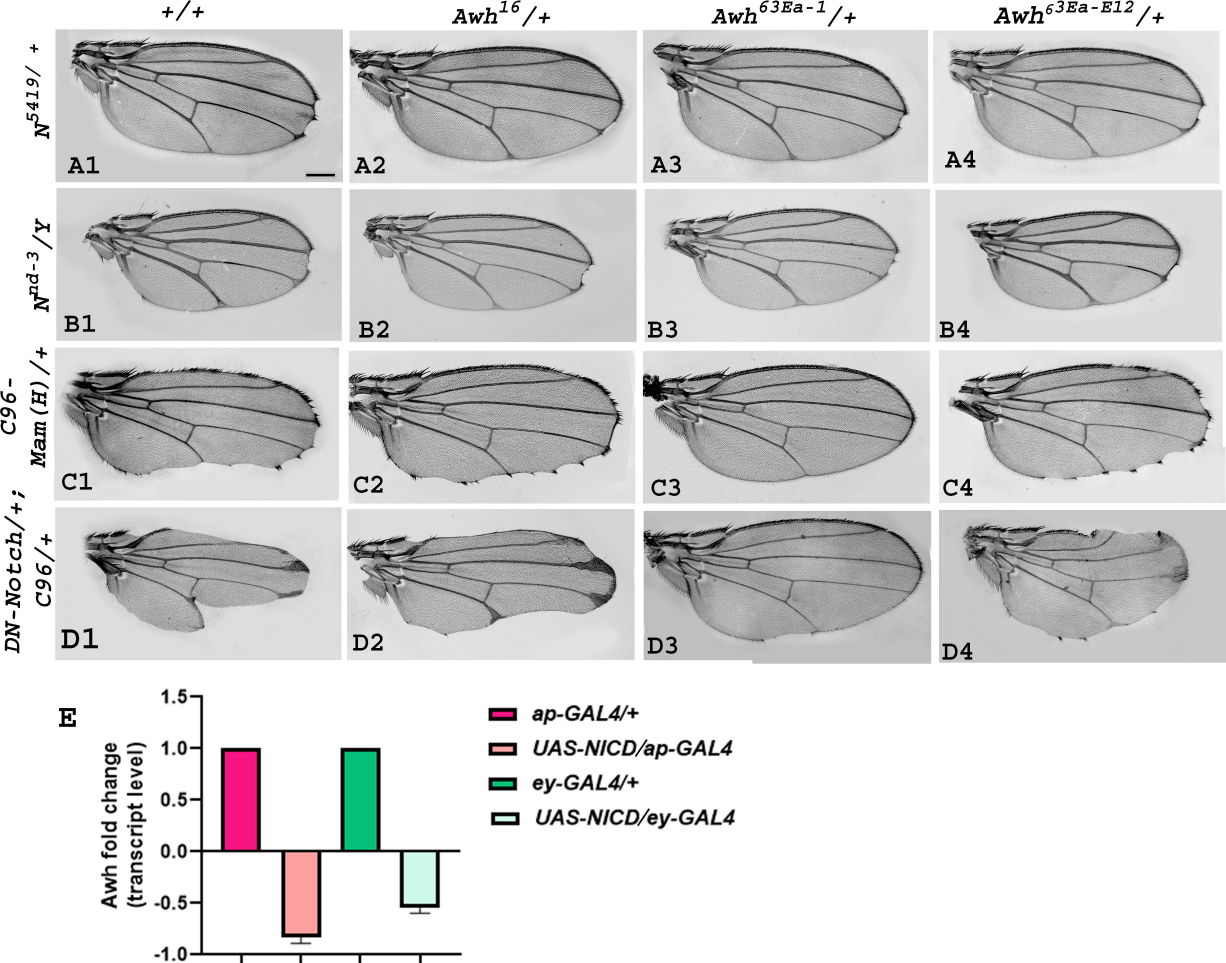
Genetic interactions of *Awh* alleles with Notch pathway components. Representative wings with genotypes are shown. Wings with *N*^*54l9*^ heterozygotes showed the wing-nicking phenotype, trans-heterozygous combination of *N*^*54l9*^ with different Awh alleles, *Awh*^*16*^, *Awh*^*63Ea-1*^ and *Awh*^*63Ea-E12*^, showed reduced wing notching (A1–A4). Notch hypomorph allele *N*^*nd3*^ showed mild notching at wing margin, whereas in trans-heterozygous combinations with *Awh* alleles, it showed rescue in wing phenotype (B1–B4). *C96-GAL4* driven dominant-negative Mastermind (*MamH*) in heterozygous condition displayed wing notching phenotype, which was reduced in trans-heterozygous combinations with *Awh* alleles; (C1–C4). Overexpression of dominant negative form of *Notch* in DV boundary exhibits severe wing notching (*DN-Notch/+; C96-GAL4/+*) (D1), which was rescued in combination with *Awh* alleles (D2–D4). A significant reduction in the level of Awh transcripts is shown in activated Notch condition using *ey-GAL4* and *ap-GAL4* through real-time PCR (E). Scale bar: 150 µm (A1-D4)

To decipher the functional implications of Awh in the Notch pathway, first, we checked whether mutations in *Awh* and Notch pathway components display genetic interaction in trans-heterozygous combinations. We used *Awh^16^, Awh^63Ea-1^,* and *Awh^63Ea-E12^* functional null alleles generated by using ethyl methane sulfonate-mediated base pair substitution [22–24]. Notch null allele *N^54l9^* showed significantly penetrant wing notching phenotype, bringing *Awh* alleles in this background in trans-heterozygous condition resulted in rescue of wing nicking, indicating enhancement of Notch function (figure 1A1–A4). Mild notching phenotype of male flies of Notch hypomorphic allele *N^nd3^* got rescued with *Awh* alleles in trans-heterozygous condition (figure 1B1–B4). Driving the dominant negative form of *Mastermind* in the C96 domain led to enhanced notching; the phenotype was rescued by reducing the level of Awh in trans-heterozygous condition (figure 1C1–C4). *C96-GAL4-*driven expression of dominant negative form of *Notch* showed fully penetrant severe wing notching. This wing-nicking phenotype of dominant negative form of *Notch* was rescued to a great extent in trans-heterozygous combination with *Awh* alleles (figure 1D1–D4). We also checked the level of Awh transcripts from wing and eye imaginal discs in which activated form of Notch was overexpressed using *ap-GAL4* and *ey-GAL4* driver strains through real-time PCR. A significant reduction in the level of Awh transcripts was observed (figure 1E). The present study provides evidence of significant genetic interaction between *Awh* and components of *Notch* pathway.

### 2.2. Loss-of-function of Awh upregulates Notch signalling

**Figure 2.**
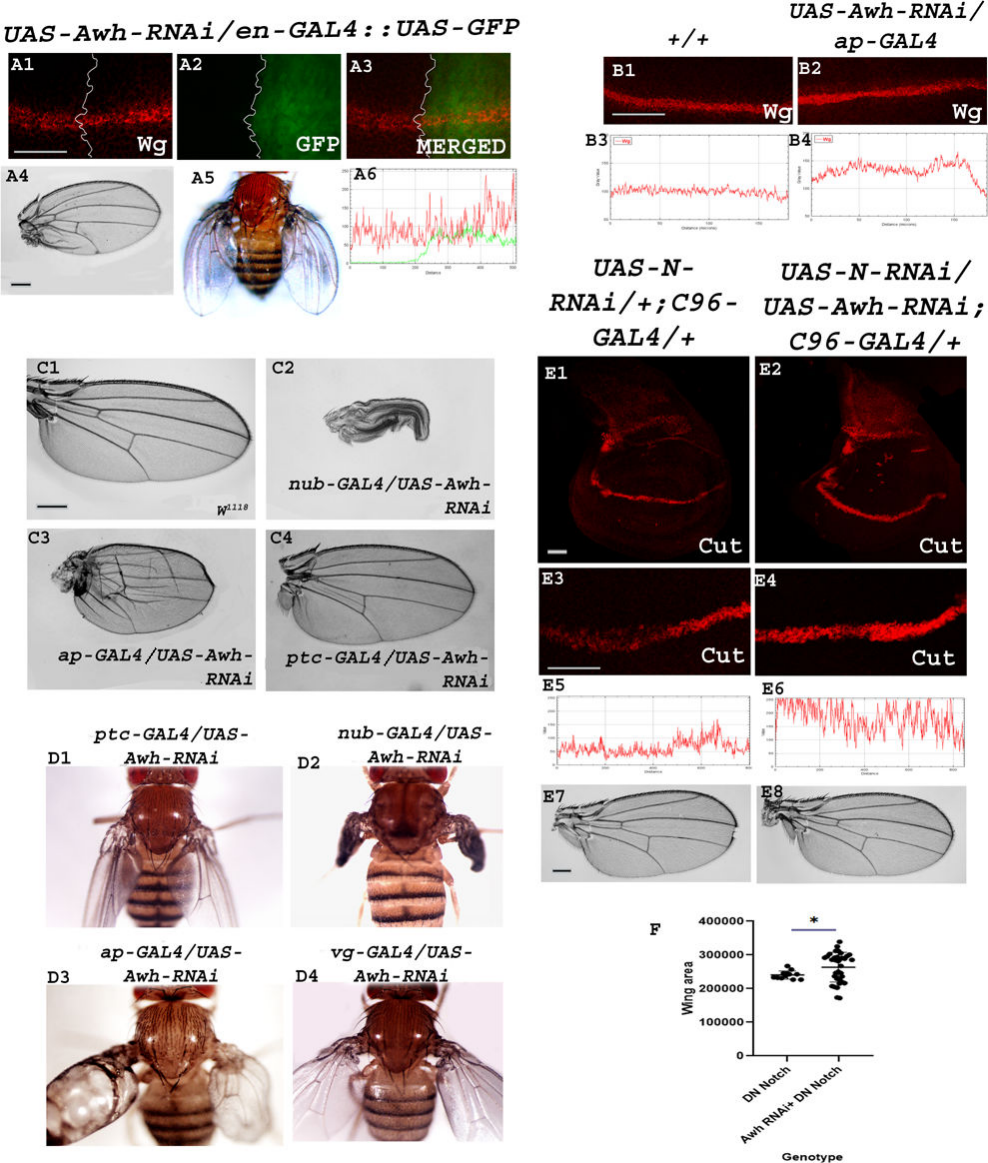
Downregulation of Awh leads to increased Notch activity. Representative wing discs showing the expression of Wg in Awh loss-of-function background using en-*GAL4*. A slight increase in Wg expression is observed in posterior compartment (A1). GFP marks the Awh downregulation domain (A2). Merged images show the Wingless expression along with Awh loss-of-function domain (A3). Adult wing showing bending of second vein, defect in vein formation with crumpling and erect wings (A4–A5). RGB profile plot shows slight increase in Wingless expression in posterior compartment (A6). Downregulation of Awh in dorsal region using *ap-GAL4* transgene led to increased expression of Wg as compared to wild-type discs (B1–B2). RGB plot profiles indicating the intensity of Wg in B1 and B2, respectively (B3–B4). Adult wing images showing the effect of Awh loss-of-function on wing development. Awh downregulation leads to paralyzed wings (*nub-GAL4*), blister formation (*ap-GAL4*), defects in vein formation, loss of anterior cross vein *(ap-GAL4* and *ptc-GAL4*) and erect wing phenotype (*ptc-GAL4, nub-GAL4, ap GAL4* and *vg-GAL4;* C1–D4). Downregulation of Notch RNAi using *C96-GAL4* leads to downregulation of Cut (E1, high magnification-E3) and notching in adult wing (E7). Bringing Awh-RNAi in the background has rescued the Cut expression (E2, high magnification-E4) and wing notching phenotype (E8). RGB profile plot shows the intensity of Cut (E5, E6). Graph shows the rescue in *DN-Notch/+; C96-GAL4*/+ wing area upon bringing *UAS-Awh* in background (F). Scale bar: 30 μm (A1–A3, B1–B2, E1–E4); 150 μm (C1–C4, E7–E8).

To decipher the loss-of-function effects of Awh, we checked the status of Notch signalling activity by investigating the expression level of downstream target of Notch, Wg in Awh loss-of-function background in third instar wing imaginal discs employing *en-GAL4* and *ap-GAL4* lines. The expression domains of Ap and En in wild-type discs are shown in electronic supplementary material, figure S1 A1–B2. Notch induces Cut and Wg expression at the dorsoventral (DV) boundary of the developing wing disc [25]. Downregulation of Awh leads to a slight increase in Wg levels as shown by immunostaining (no. of discs examined—20 each). Awh loss-of-function in posterior domain leads to small wing with bending of second vein, extra vein material in fourth and fifth vein and crumpling in posterior region. The adult fly shows erect wings. Red, green, blue (RGB) profile plots display the increased fluorescence intensity of Wg in the regions where Awh was downregulated (figure 2A1–B4).

Downregulation of Awh in the pouch region leads to formation of paralyzed wing where the wings fail to open up. Its loss-of-function in dorsal region using *ap-GAL4* transgene leads to blister formation, severe defects in wing vein formation, extra vein material at hinge region and severe crumpling. Similarly, Awh downregulation in AP boundary leads to increased vein material between third and fourth vein and loss of anterior cross vein. It is interesting to notice that Awh downregulation in AP boundary, wing pouch and dorsal and ventral region of wings leads to erect wing phenotype (figure 2C1–D4).

Furthermore, we checked the effect of Awh downregulation in Notch downregulation background. Downregulation of Notch in DV boundary using RNAi line leads to decreased expression of Cut in wing imaginal disc and notching at wing margin in adults (28% of flies show notching; no. of flies observed = 80). Interestingly, upon bringing Awh loss-of-function in the background, the Notch loss-of-function, the Cut expression was rescued and no notching was observed in adults (no. of flies observed = 80; figure 2E1–E8). Similar experiment was performed using dominant negative form of Notch. It was observed that there was a rescue in wing size upon bringing Awh-RNAi in *UAS-DN-Notch/+; C96-GAL4/+* background (figure 2F). From the dataset, it is evident that Awh loss-of-function has significantly rescued Notch loss-of-function phenotype.

### 2.3. Awh downregulates Notch signalling

**Figure 3.**
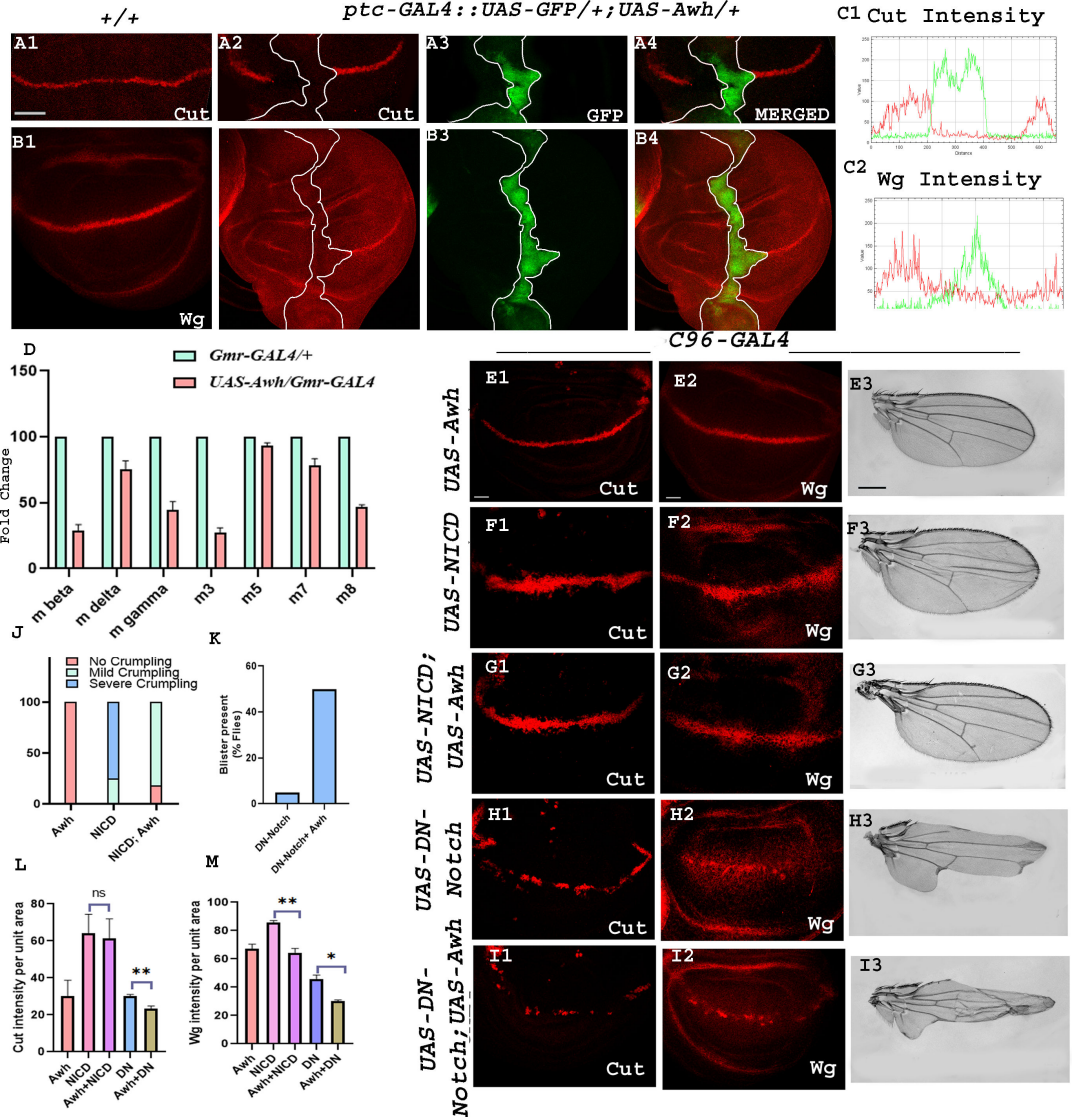
Downregulation of Notch signalling by Awh overexpression. Overexpression of Awh in AP boundary using *ptc-GAL4::UAS-GFP* transgene led to a significant reduction in level of Cut and Wingless as compared to wild-type wing discs. GFP marks the Awh overexpression domain. Merged images show Cut and Wg expression along with GFP. White line marks the boundary of Awh overexpression (A1–B4). RGB plot profiles indicating the intensity of A4 and B4, respectively. Red marks the Cut and Wg intensity, and green marks the Awh overexpression domain (C1, C2). Level of Notch target, *E(spl)* complex gene transcripts (*mβ, mγ, m∆, m3, m5, m7, m8*) are shown in Awh overexpression background; *GAL4* control was used. Reduction in all seven *E(spl)* complex gene transcripts was observed in Awh overexpression condition (D). Cut and Wg expression is shown in C96-driven Awh overexpression condition (E1, E2). NICD overexpression with *C96-GAL4* showed increased Cut and Wg expression in wing discs (F1, F2) and crumpled wings in adults (F3). Co-expression of Awh and NICD rescue the Cut and Wg expression to some extent (G1, G2); rescue in wing crumpling was also observed (G3). Graph showing the ratio of mild and severe crumpling in wings with different combinations of Awh and NICD (J). Overexpression of dominant negative form of Notch in DV boundary showed significant reduction in level of Notch targets Cut and Wg (H1, H2). Adult with overexpressed dominant-negative Notch showed severe notching (H3). Overexpressing Awh together with dominant negative form of Notch led to significant disruption in targets Cut and Wg (I1, I2) as well as in adult phenotype (I3) as compared to Notch-DN. The adult wing also displays increase in notching and presence of blisters (H1–I3). Graph showing the increase in percentage of flies with blister (K). Intensity profiling of Cut and Wg was shown in different combinations of Awh, NICD and DN-Notch driven with *C96-GAL4* (L, M). Scale bar: 30 μm (A1–B4, E1–E2, F1–F2, G1–G2, H1–H2, I1–I2); 150 µm (E3, F3, G3, H3, I3).

To explore the involvement of Awh in Notch pathway we checked the status of Notch signalling activity by investigating the expression levels of downstream targets of Notch, Cut and Wg in Awh overexpressed wing imaginal discs. Overexpression of Awh by *ptc-GAL4* in anterior–posterior (AP) boundary showed complete reduction of Notch targets, Cut and Wg at the AP and DV intersection in comparison to wild-type control as shown in images (figure 3A1–B4; no. of discs examined = 30). RGB profile plots display the decreased fluorescence intensity of Cut and Wg in the regions with overexpressed Awh compared to the internal control (figure 3C1, C2). Electronic supplementary material, figure S1 C1, C2 shows the expression domain of Ptc in wild-type discs. In addition, we checked the transcript levels of Notch targets, *E(spl*) complex genes in Awh overexpressed conditions using real-time PCR. *E(spl*) contains seven transcription units *mβ*, *mγ*, *mδ ,m3*, *m5*, *m7* and *m8* that encode basic helix–loop–helix containing transcription factors [26]. The expression of all seven E(spl)- HLH transcripts was downregulated in Awh overexpression background in comparison to *Gmr-GAL4/+* control (figure 3D).

Furthermore, we checked the loss and gain-of-function effects of Notch on the Awh overexpressed wing imaginal discs. As modulation in Notch levels using *ptc-GAL4* leads to embryonic lethality, *C96-GAL4,* a comparatively weak GAL4 transgene was used for interaction studies. Electronic supplementary material, figure S1 D1 and D2 shows the expression domain of *C96-GAL4* in wild-type discs. Awh overexpression in the DV boundary using *C96-GAL4* resulted in mild defects in bristle patterning while no significant changes were observed in Cut and Wg expression (figure 3E1–E3). Overexpression of NICD alone in DV boundary led to increased expression of Notch targets, Cut and Wg levels, hence consequently leading to mild crumpling and irregular bristle patterning in adult wing, whereas co-expressed *UAS-Awh* along with *UAS-NICD* in DV boundary showed decrease in wing size and crumpling of adult wing, as shown in graph (no. of wings examined = 80; figure 3J). The levels of Notch targets Cut and Wg were also rescued in the co-expression background (no. of discs examined = 30; figure 3F1–G3). Furthermore, we checked the interaction between Awh and Notch dominant negative. Dominant negative form of Notch is the Notch receptor lacking Notch activation domain (NICD) [27]. Overexpressing Notch dominant negative in *C96-GAL4* domain led to severe wing notching. Co-expression of Awh and dominant negative form of Notch further worsens the wing phenotype; the severity of notching increases, and presence of blisters was also observed in 50% of flies, as shown in graph (no. of wings examined = 80; figure 3K). This prompted us to check the level of Notch targets in this background, and we noticed that the levels of Cut and Wg were significantly downregulated in *UAS-DN-Notch; UAS-Awh/ C96-GAL4* in comparison with *UAS-DN-Notch/+; C96-GAL4/+* (no. of discs examined = 30; figure 3 I1-I3). This result showed that Awh downregulates Notch signalling. Quantification of Notch targets, Cut and Wg, was done in the same background. About three discs were used for quantification using ImageJ software where integrated density/area of the domain was used for quantification purposes. Unpaired *t*‐test was performed to determine the significance of our findings. Intensity profiling further validated the role of Awh in downregulation of Notch signalling (figure 3L,M).

### 2.4. Awh overexpression modulates Wingless signalling

**Figure 4.**
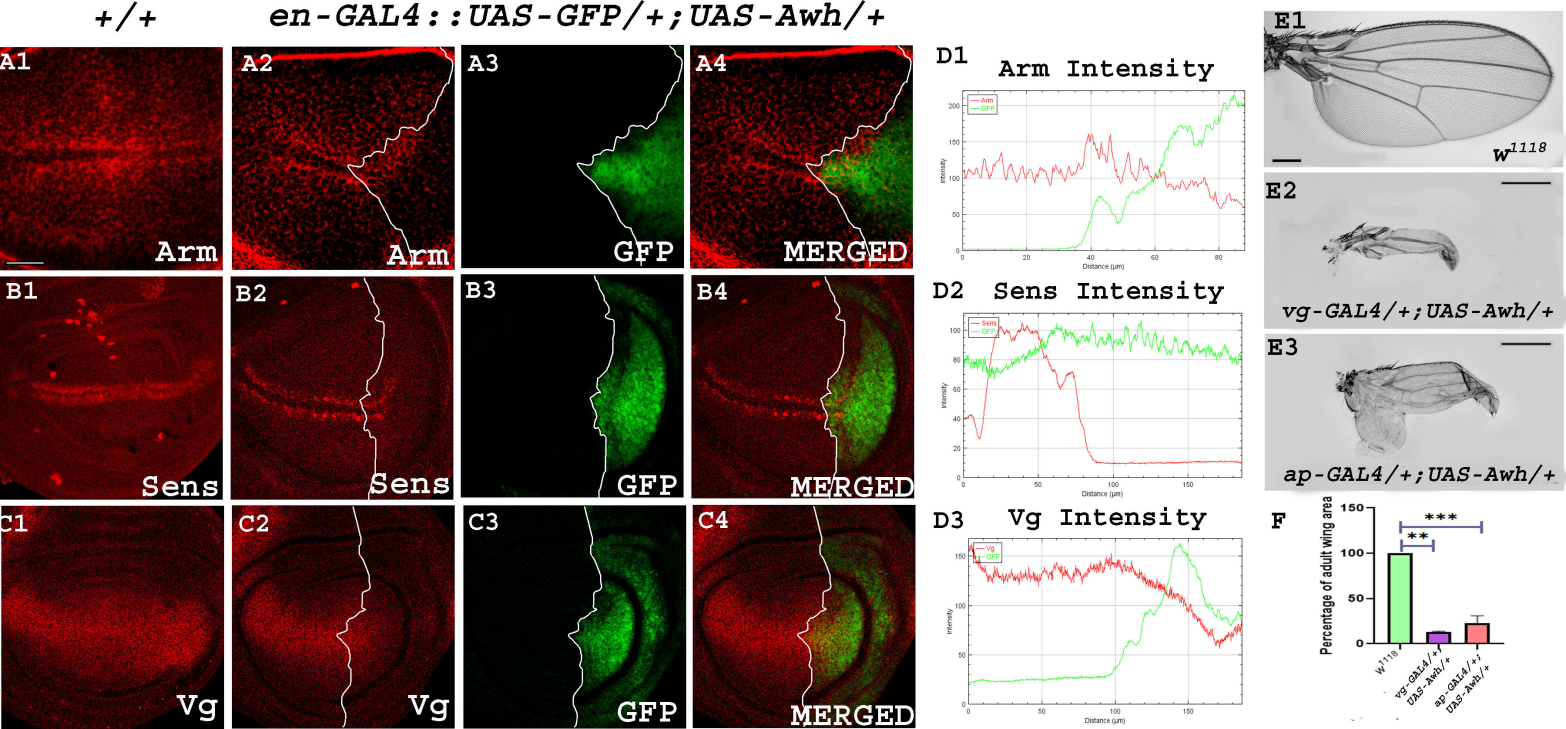
Awh modulates the expression of Wg signalling components. Representative wing disc shows Arm expression in wild-type control discs (A1). Awh overexpression in the posterior domain of wing disc using *en-GAL4* led to significant disruption in Arm expression. GFP marks the posterior domain of the third instar wing disc. Merged image shows the expression of Arm and GFP in Awh overexpression condition. White line marks the boundary of Awh overexpression (A2–A4). Representative wing disc shows Sens expression in wild-type control discs (B1). Awh overexpression led to decrease in Sens expression in posterior compartment. GFP marks the posterior domain of the third instar wing disc. Merged image shows the expression of Sens and GFP in Awh overexpression background. White line marks the boundary of Awh overexpression (B2–B4). Representative wing disc shows Vg expression in wild-type discs (C1). Awh overexpression in posterior compartment downregulated Vg expression. GFP marks the posterior domain of the third instar wing disc. Merged image shows expression of Vg and GFP in Awh overexpression. White line marks the boundary of Awh overexpression (C2–C4). RGB plot showing intensity of Arm, Sens and Vg in Awh overexpression. Red marks the intensity of Arm. Sens and Vg and green mark the Awh overexpression domain (D1–D3). Representative wings with genotypes are shown. Awh overexpression in ventral and dorsal regions by *vg-GAL4* and *ap-GAL4* (E2, E3) led to small adult wings in comparison to wild-type wings (E1). Graph showing the comparison of adult wing area in control and Awh overexpression wings (F). Scale bar: 30 μm (A1–C4); 3 cm (E1–E3).

Since Awh overexpression led to the downregulation of Notch target Wg, we checked the effect of Awh overexpression on Wg signalling activity and wing development. Wg plays a central role in pattern formation during development [15]. In wild-type tissue when Wg binds to receptor Frizzled, it leads to the stabilization of Arm protein; this stabilized Arm enters the nucleus and activates downstream target genes: *sens, dll and vg* [15,28,29]. In the absence of ligand Wg, the multiprotein destruction complex, APC, Axin and GSK-3, degrades Wg effector, Arm by phosphorylation [28]. Interestingly, overexpression of Awh in posterior compartment of third instar wing discs showed a strong disruption in the level of Arm that consequently led to the downregulation of short-range and long-range targets Sens and Vg in comparison to the wild-type discs (no. of discs examined = 30; figure 4A1–C4). RGB plot shows a significant downregulation of Wg targets in the domain of Awh overexpression (figure 4D1–D3). Furthermore, Awh gain-of-function mediated changes in adult wing were observed. *vg-GAL4* and *ap-GAL4* driven Awh overexpression in ventral and dorsal regions of *Drosophila* wing discs, respectively, forms vestigial wing phenotype reminiscent of wingless and vestigial loss-of-function phenotype that further strengthens our study (no. of wings examined = 80; figure 4E1–E3) [30]. Quantitative analysis of adult wing size was performed using ImageJ software, and the graph was plotted using Graph Pad Prism 8 software. Statistical analysis highlights the significant reduction in wing size in Awh overexpression background (figure 4F).

We also checked whether the influence of Awh on Wg signalling is Notch-mediated. To this end, activated form of Notch was provided in Awh gain-of-function background, and the expression of Wg was observed. Temperature-sensitive *ptc-GAL4::UAS-GFP* was used for our study. As *ptc-GAL4*-mediated NICD overexpression is embryonic lethal, temperature-sensitive GAL80 system was employed for temporal regulation of the UAS transgene. As expected, Awh gain-of-function completely abolished Wg expression in the AP domain. Overexpression of NICD led to increased expression of Wg along the AP boundary. When *UAS-Notch-ICD* and *UAS-Awh* were co-expressed, Wg expression was significantly reduced in wing discs compared to that of only NICD overexpressed wing imaginal discs. This result indicated that Awh-regulated Wg signalling might be controlled through Notch pathway (electronic supplementary material, figure S2).

#### 2.4.1. Awh regulates wing patterning

**Figure 5.**
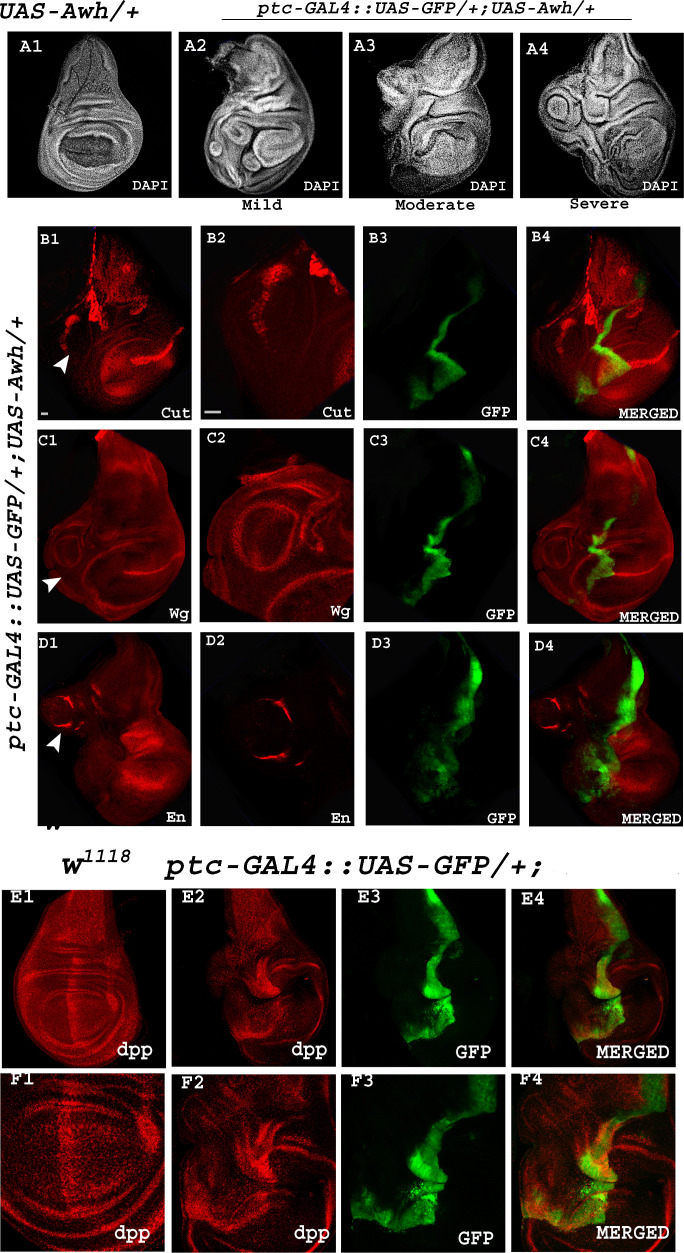
Awh regulates wing patterning. Awh overexpression in AP boundary using *ptc-GAL4::UAS-GFP* leads to drastic defects in wing discs morphology in comparison to control discs as shown by DAPI stain (A1). Defects were categorized as mild, moderate and severe (A2–A4). Representative wing disc showing ectopic expression of Cut, Wg and En in the anterior compartment of third instar wing disc in Awh overexpression condition (B1, C1, D1, high magnification B2, C2, D2). GFP marks the domain of Awh overexpression (B3, C3, D3). Merged images show expression of Cut and GFP, Wg and GFP, En and GFP (B4, C4, D4). Overexpression of Awh in AP boundary leads to significant disruption in dpp expression (E2–E4; high magnification F2–F4) as compared to wild-type discs (E1, F1). Scale bar: 30 μm (A1–D4, E1–F4).

Awh overexpression in the AP boundary of wing discs using *ptc-GAL4* driver leads to several types of patterning defects in wing discs. In total, 70% of wings show drastic inward folding of wing imaginal discs, while 15% of discs manifest small duplication and 15% show complete duplication in the wing pouch (no. of discs examined = 60; figure 5A2–A3). We checked the expression of Cut and Wg in the duplicated region. The duplicated part showed ectopic Cut and Wg expression (no. of discs examined = 8; figure 5B1,C1, high magnification in B2, C2). Clonal experiments were performed to interrogate the spatial regulation of Awh gain-of-function-mediated wing duplication. We found that the clone specifically formed in the dorsal compartment of wing disc showed ectopic expression of Cut and Wg. The clones of the ventral region showed no change in expression of these Notch targets, while clones formed on the DV boundary region showed loss of Cut and Wg (no. of discs examined = 20; electronic supplementary material, figure S3). Earlier studies have shown that the expression pattern of Awh transcripts in stages 14−16 of embryo is very much similar to segment polarity genes [22]. Segment polarity genes, *wg* and *engrailed* (*en*), play profound roles in wing development [15,31]. In this context, along with Wg expression, we also checked En expression and found En is also ectopically expressed in duplicated wing pouch area (no. of discs examined = 8; figure 5D1, high magnification in D2) in Awh overexpressed wing imaginal discs. This kind of duplication and ectopic expression of Wg and En clearly indicates the critical role of Awh in wing development.

Furthermore, we observed that Awh overexpression-mediated wing disc defects were outside the *ptc-GAL4* domain indicating its non-cell autonomous effect. This observation prompted us to check whether Awh can alter the expression of any secreted disc mitogen. At this end, we checked the expression of dpp, a TGF-beta homologue. Dpp is a ligand that specifies cell fate along the AP axis of the adult wing. It acts as a morphogen as well as mitogen [32,33]. Overexpression of Awh in AP boundary shows significant disruption in expression of Dpp. In Awh overexpression condition, the expression of dpp stripe at DV boundary also gets broadened compared to its expression in wild-type discs. Similarly, the expression of Ptc stripe at AP boundary also gets widened as shown in figure 5E3,F3 as compared to Ptc wild-type expression (electronic supplementary material figure S1 C1, C2). These results indicate that Awh-mediated wing patterning defects are non-cell autonomous effect and are mediated via secreted mitogen such as Dpp.

#### 2.4.2. Right amount of Chip expression is crucial for Awh function

It has been shown earlier that Awh prevents retinal differentiation and promotes differentiation to ventral head tissue in the regions of eye disc, and Awh needs transcriptional co-factor Chip for this function [34]. We have shown earlier that Chip interacts with Notch, and it plays a major role in Notch-induced DV margin formation and cell proliferation [35]. These observations prompted us to check the role of Chip in Awh-mediated wing development.

**Figure 6.**
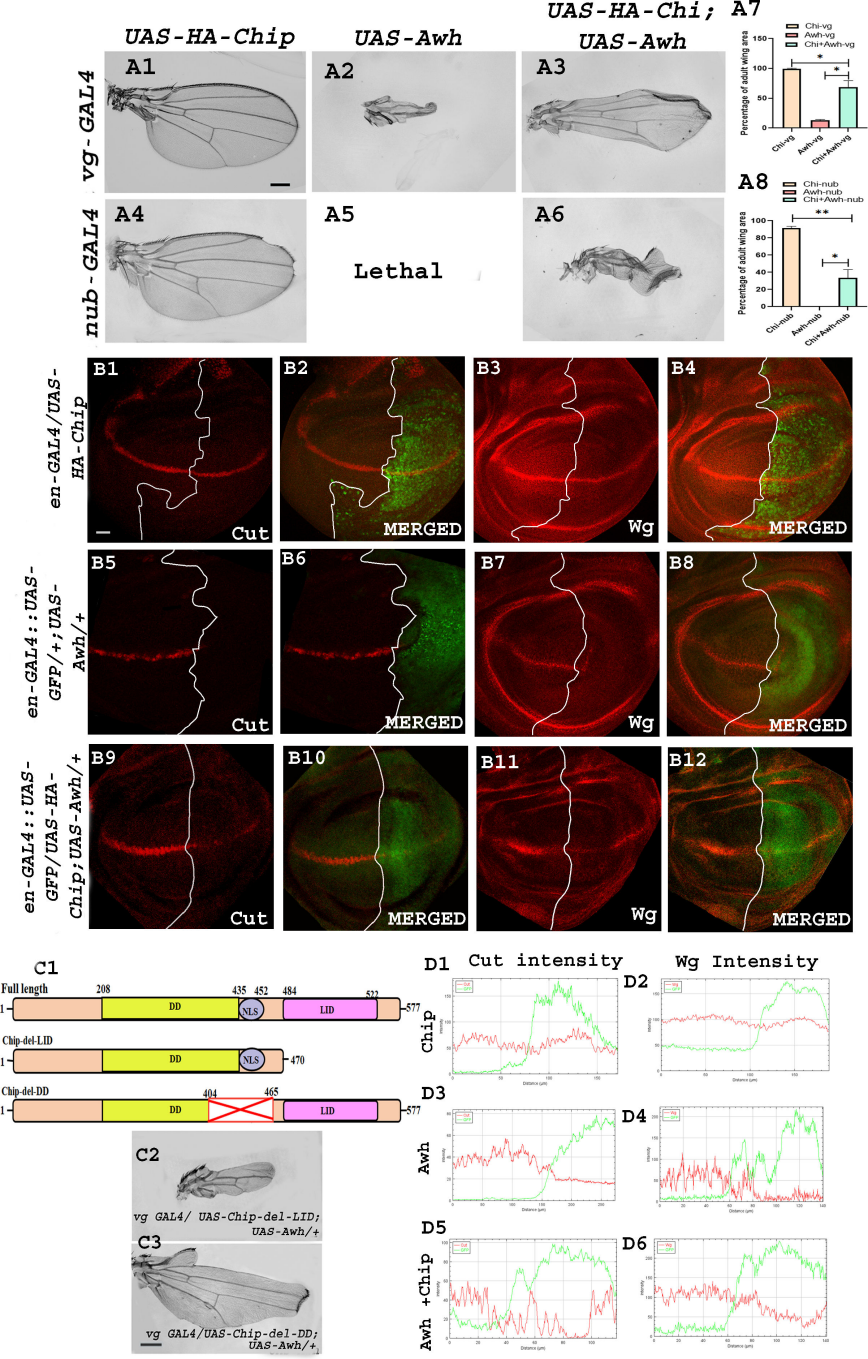
Chip rescue Awh gain-of-function phenotype. Representative wings with genotypes are shown. Chip overexpression in the ventral region of the wing showed no significant change (A1). Awh overexpression in the ventral region showed severe loss of wing tissue (A2), while co-expression of Chip and Awh rescued the wing phenotype (A3). Chip overexpression in pouch region shows mild notching (A4). Awh overexpression using *nub-GAL4* led to pupal lethality (A5), while co-expression of Awh and Chip in pouch region rescued the lethality, and the adult wing showed severe crumpling (A6). Graph showing the rescue of wing area in Awh and Chip co-expression (A7–A8). Representative wing disc showing expression of Cut and Wg in HA-Chip overexpression in posterior compartment (B1, B3). White lines show the boundary, and green stain shows the region of Chip overexpression (B1–B4). Expression of Cut and Wg was completely lost in Awh gain-of-function condition (B5, B7). White lines show the boundary and GFP marks the region of Awh overexpression (B5–B8). Co-expression of Chip and Awh restores the expression of Cut and Wg to some extent (B9, B11). White lines show the boundary, and GFP shows the domain of Awh and Chip co-expression (B9–B12). RGB profile plot shows intensity of Cut and Wg in different genetic combinations. Red marks the Cut, Wg intensity, and green marks the domain of overexpression (D1–D6). Domain structure of full-length Chip and deletion lines are shown (C1). Overexpressing Chip with deleted LIM domain along with Awh showed no recovery in wing size. Overexpressing Chip with deleted dimerization domain along with Awh showed recovery in wing size (C2–C3). Scale bar: 150 μm (A1–A6, C2–C3); 30 μm (B1–B12).

*vg-GAL4*-driven Awh overexpression in the ventral domain of the wing discs displayed severe loss of wing tissue, whereas the co-expression of Awh and Chip in the ventral domain of wing discs rescued this wing phenotype. Similarly, Awh overexpression in the pouch region using *nub-GAL4* led to pupal lethality, whereas the co-expression of Awh and Chip led to rescue from pupal lethality (no. of wings examined = 50; figure 6A1–A6), which showed that the right amount of Chip is important for proper Awh function. Graphical representation of the percentage of wing area showed significant recovery of adult wing size in Awh and Chip co-expression (figure 6A7–A8). We also observed recovery in wing disc size and morphology in Awh and Chip co-expression background (electronic supplementary material, figure S4). Similarly, we found that Awh overexpression in the posterior region of the wing imaginal discs by *en-GAL4* led to abolition of Notch targets Cut and Wg, while the co-expression of Awh and Chip in posterior domain restored the Cut and Wg (no. of discs examined = 25; figure 6B1–B12) levels. RGB profile plots highlight the similar observations (figure 6D1–D6). Thus, it is evident again that a critical amount of Chip is important for Awh function.

Chip contains a self-dimerization domain and a LIM binding domain. In order to check which domain of Chip is crucial for interaction with Awh, different Chip deletion lines were co-expressed with Awh. Two deletion lines of Chip were used; one line has deleted the LIM binding domain, while the other one has deleted the dimerization domain. Different domains of full-length Chip protein and the deletion lines are shown in figure 6C1. Bringing Awh in combination with Chip deleted LIM binding domain led to no significant rescue, while its co-expression with Chip deleted dimerization domain led to a remarkable rescue in adult wing phenotype (no. of wings examined = 80; figure 6C2,C3). This dataset suggests to us that the LIM binding domain of Chip is crucial for Awh functioning.

### 2.5. Awh regulates Notch-mediated neuronal defects

**Figure 7.**
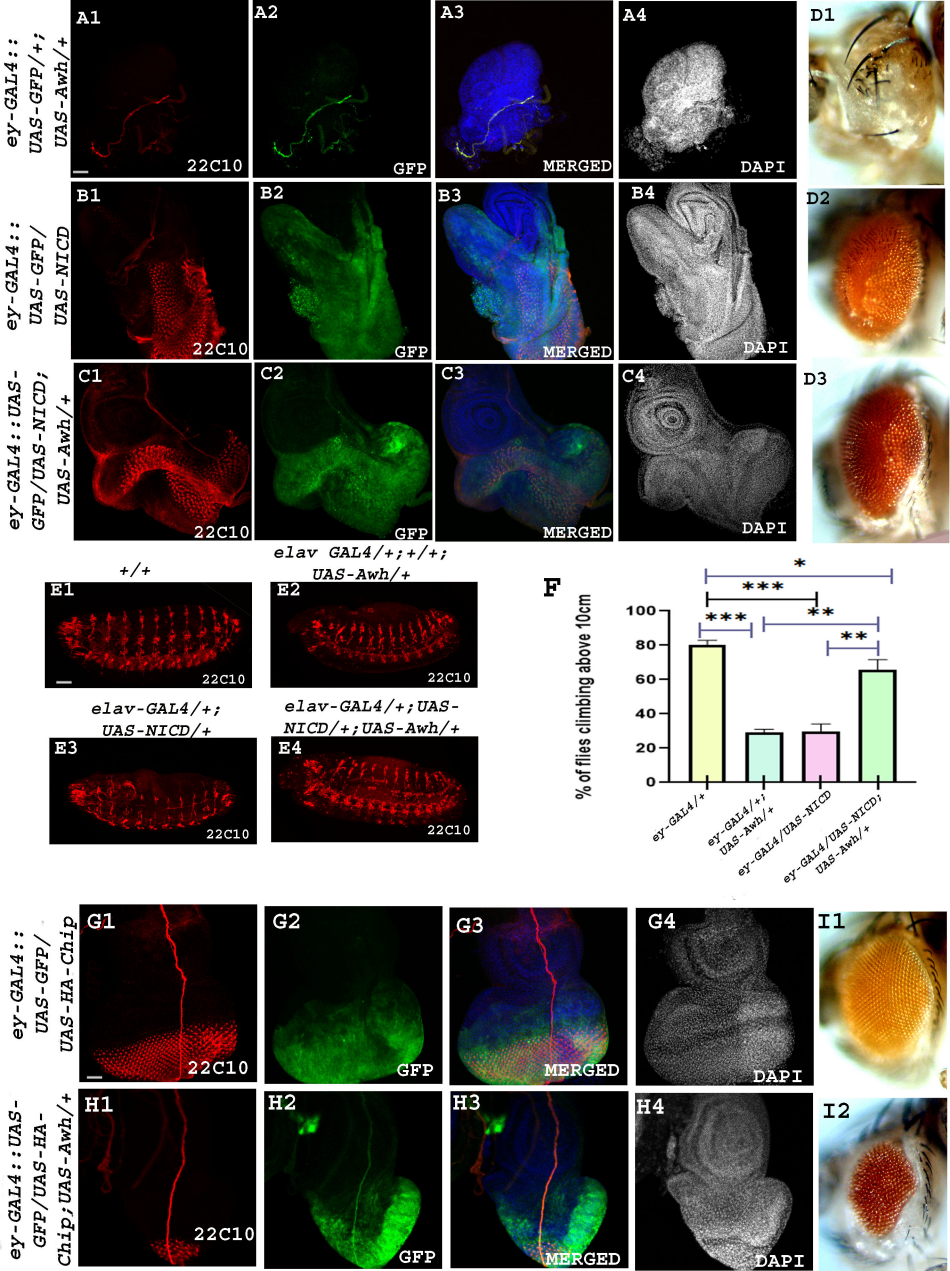
Awh regulates Notch-mediated neuronal defects. Overexpression of Awh using *ey-GAL4* led to loss of neuronal tissue marked with 22C10 (A1). GFP marks the domain of Awh overexpression (A2). Merged image shows 22C10, GFP and DAPI (blue) together. DAPI shows eye disc morphology (A3, A4). Overexpression of NICD using *ey-GAL4* led to disrupted 22C10 expression (B1). GFP marks the domain of NICD overexpression (B1). Merged image shows 22C10, GFP and DAPI (blue) together. DAPI shows hyperproliferative eye disc (B3, B4). Co-expression of Awh and NICD using *ey-GAL4* led to restoration of 22C10 expression (C1). GFP marks the co-expression domain (C2). Merged image shows 22C10, GFP and DAPI (blue) together. DAPI shows recovery of eye morphology (C3, C4). Representative adult eye showing ablated eye in Awh overexpression condition (D1). NICD overexpressed adult shows hyperproliferative eye (D2), while the adult eye shows rescue in Awh and NICD co-expressed background (D2). Similar results were observed in embryos using the 22C10 antibody. Overexpression of NICD using *elav-GAL4* showed significant defects in the ventral nerve cord of stage 14 embryo in comparison to wild-type embryo. Co-expression of Awh and NICD led to significant rescue of ventral nerve cord (E1–E4). Climbing ability was monitored using climbing assay. Only Awh and only NICD overexpressed flies showed defect in climbing ability, while co-expression of Awh and NICD showed significant rescue in the climbing ability (F). Co-expression of Chip and Awh using *ey-GAL4* rescued the Awh overexpression-mediated photoreceptor cell defects shown by 22C10 (H1). GFP marks the co-expression domain (H2). Merged image shows 22C10, GFP and DAPI (blue) together. DAPI shows recovery of eye morphology (H3, H4). Scale bar: 30 μm (A1–C4, E1–E4, G1–H4).

Earlier the role of Awh in neuronal development was shown. Awh overexpression using *Da-GAL4* leads to defects in the Futsch expression pattern [21]. Futsch is a microtubule-binding protein that helps in formation of synaptic buttons at neuromuscular junctions [36]. Notch being identified as a neurogenic gene plays a crucial role in neurogenesis and neuron maintenance. To study the functional application of Awh–Notch regulation, we checked the level of neuronal marker, Futsch/22C10, in different combinatorial manner. Increasing the level of Awh in the eye discs using *ey-GAL4* caused significant loss of neuronal cells as well as loss of eye tissue in adults. On the contrary, NICD overexpression in the eyeless region led to disruption in the Futsch pattern and proliferative eye phenotype in adults. The combinatorial effect of Awh and activated Notch in the eye led to betterment of eye phenotype, and the pattern of neuronal marker 22C10 also got restored (figure 7A1–D3). Similar results were obtained in *Drosophila* embryos stage 14 using *elav-GAL4* (figure 7E1–E4). To further validate the study, the climbing ability of different sets of flies was observed. Only Awh and activated Notch flies showed defects in their climbing ability while bringing them together restored their climbing behaviour in comparison to GAL4 control flies (no. of flies examined = 50; figure 7F).

Furthermore, we explored the effect of Chip on Awh-mediated neuronal defects. Overexpression of Chip in eyeless domain shows normal pattern of photoreceptor cells. Co-expressing Awh with Chip rescues Awh-mediated loss of photoreceptor cells (no. of disc examined = 25). A prominent rescue is also observed in adult eye (figure 7G1–I2).

### 2.6. Awh regulates Notch signalling by affecting Delta expression

**Figure 8.**
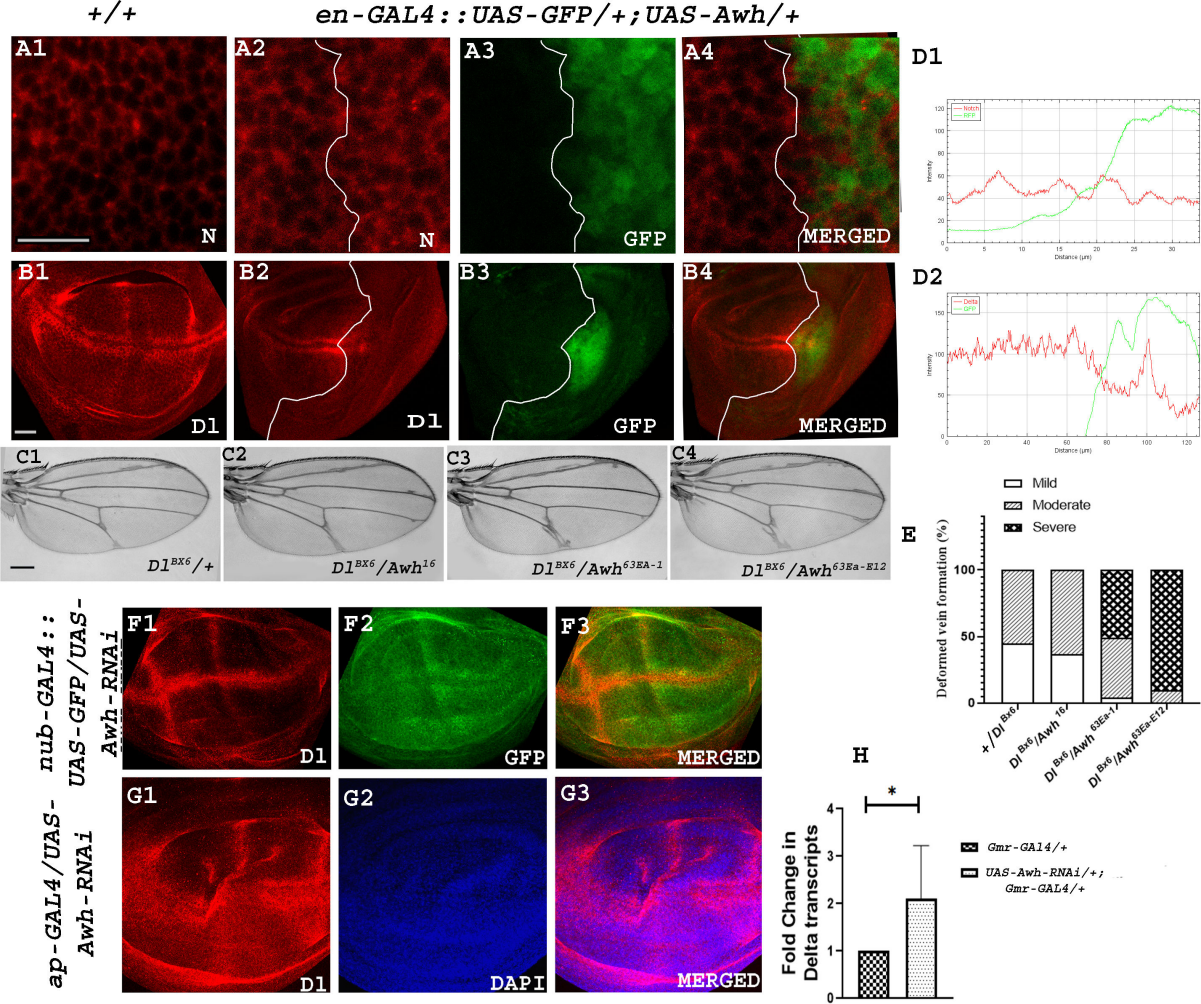
Awh affects Notch pathway by regulating Delta expression. Representative wing disc showing Notch receptor expression in wild-type wing discs (A1). Awh overexpression in the posterior domain of wing disc using *en-GAL4:: UAS-GFP* led to no change in expression of Notch receptor. GFP marks the posterior domain of the third instar wing disc. Merged image shows the expression of Notch and GFP in Awh overexpression. White line marks the boundary of Awh overexpression (A2–A4). Representative wing disc showing Delta expression in wild-type wing discs (B1). Awh overexpression in the posterior domain of wing disc using *en-GAL4* led to significant reduction in Dl expression. GFP marks the posterior domain of the third instar wing disc. Merged image shows the expression of Dl and GFP in Awh overexpression. White line marks the boundary of Awh overexpression (B2–B4). RGB profile plot demonstrates the intensity of Notch and Dl (marked with red) and Awh overexpression domain (marked with green; D1–D2). Representative wings with genotypes are shown. *Dl^BX6^* showed vein-thickening phenotype; the thickening was significantly enhanced upon bringing Awh functional null *Awh^16^, Awh^63Ea-1^* and *Awh^63Ea-12^* in the background (C1–C4). Graph shows the percentage of flies with mild, moderate and severe level of vein thickening in different combinations (E). Awh downregulation in pouch region using *nub-GAL4:: UAS-GFP* shows increased Delta expression (F1). GFP marks the domain (F2). Merged image shows the expression of Delta and domain of Awh downregulation. Delta expression in Awh loss-of-function using *ap-GAL4* is shown (G1). DAPI marks the disc morphology (G2). Merged images show expression of Delta and DAPI (G3). Graph shows increase in Delta transcripts in Awh loss-of-function background (*p* value: 0.0354; H). Scale bar: 30 μm (A1–B4, F1–G3); 150 μm (C1–C4).

Since overexpression of Awh led to reduced expression of Notch targets, we wanted to dissect its effect on the status of Notch receptor. We checked the level of endogenous Notch protein in the wing discs in which Awh was overexpressed in the posterior compartment using *en- GAL4* transgene. Notch expression and localization remained unaltered in the Awh overexpressing domain of the wing disc (figure 8A1–A4). This shows that Awh downregulates Notch signalling without affecting Notch receptor levels.

As the presence and binding of DSL ligands is crucial for Notch receptor activation [3] and Delta is already shown to regulate Awh transcripts [22], it prompted us to check the level of Delta in Awh overexpressed discs. The expression pattern of Delta was analysed while overexpressing Awh in the posterior compartment of the third instar wing disc. Awh gain-of-function illustrates a remarkable decrease in Delta in the posterior compartment (figure 8B1–B4). Further genetic interaction studies were performed to fortify our observation. *Delta* alleles showed vein -thickening phenotype. The severity of vein thickening was further enhanced upon bringing *Awh* alleles in background confirming the genetic interaction between the molecules (figure 8C1–C4). Wings were categorized into mild, moderate and severe with different degree of vein thickening. Scoring of the adult wing was done. *Dl^BX6^* shows wings with 45% mild and 55% moderate vein thickening. Bringing Awh allele, *Awh^16^, Awh^63Ee-1^* and *Awh^63Ea-12^*, led to mild vein thickening to 37%, 4%, 0% moderate vein thickening to 63%, 45% and 9.5% and severe vein thickening in 0%, 50%, 90.5% of wings, respectively (figure 8E; no. of wings observed = 50). This study suggests that Awh-mediated Notch signalling downregulation is Delta-mediated.

To strengthen our hypothesis, we analysed the level of Dl in Awh loss-of-function condition. Awh was downregulated in the entire wing pouch using *nub-GAL4* and checked the expression of Delta. It was observed that there was an increase in Dl level (figure 8F1). Similarly, downregulation of Awh in dorsal compartment leads to a significant disruption in Dl expression (figure 8G1). We also checked the level of Dl transcripts upon downregulating Awh in eye imaginal disc using *Gmr-GAL4* transgene. An increase in Delta transcripts was observed in Awh downregulation condition (figure 8H). This dataset confirms that there is a negative feedback loop between Awh and Delta.

### 2.7. Awh activity is regulated by activated Notch

**Figure 9.**
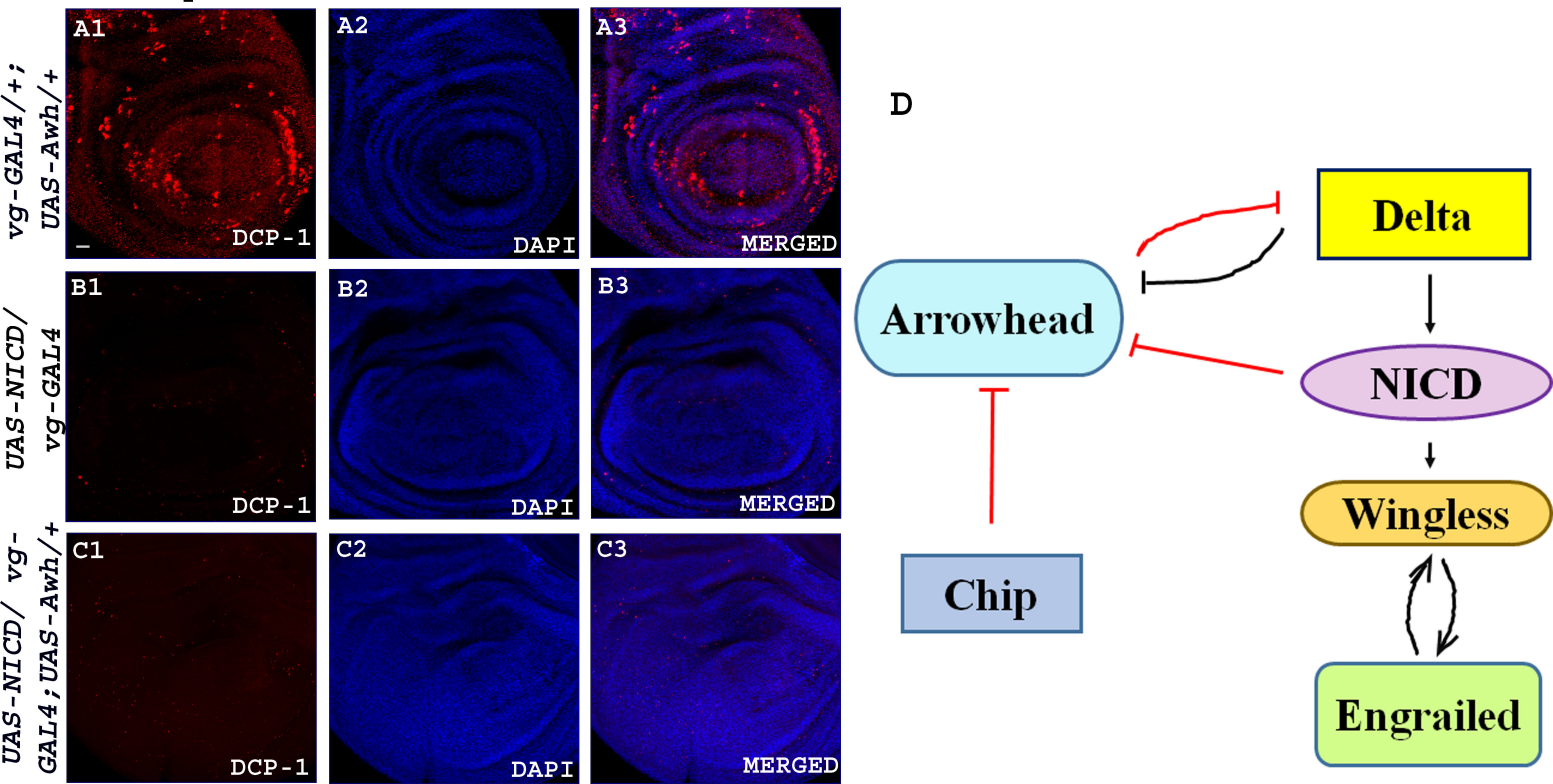
Awh activity is inhibited by activated Notch. Representative wing disc showing expression of dcp1-1 in Awh overexpression in the ventral region; morphology of disc was visualized with DAPI (A1–A3). NICD overexpression in ventral region showed no expression of dcp-1. DAPI shows the morphology of the disc (B1–B3); Awh and NICD co-expression also showed no expression of dcp-1. DAPI shows the morphology of the disc (C1–C3). The model shows the summary of the present work. The red arrows mark the novel finding, and the black arrows are the established interactions (D). Scale bar: 30 μm (A1–C3).

Awh is reported to cause programmed cell death and loss of adult structures in overexpressed condition [22]. We also observed increased acridine orange positive cells that marked the dying cells when Awh was overexpressed in different domains of wing discs (electronic supplementary material, figure S5). It was seen that Awh-mediated cell death is caspase-dependent (electronic supplementary material, figure S6). To determine whether this Awh-mediated Notch signalling downregulation is caspase-dependent or not, we checked the level of Notch target Wg upon bringing cell-death inhibitor *UAS-p35* in Awh overexpression background. Inhibition of caspase-mediated cell death shows recovery in size of wing discs, but no significant rescue was observed in Wg expression at DV boundary. Similarly, the adult also shows some recovery in wing size, but no recovery was observed in wing margin confirming that Awh gain-of-function-mediated Notch regulation is independent of cell death (electronic supplementary material, figure S7). *vg-GAL4*-driven overexpression of Awh transgene in the ventral region shows increased expression of dying cells marked by DCP-1. Earlier it has been shown that overexpression of *NICD* in the ventral region shows a hyper-proliferative phenotype with no cell death in the wing disc [37]. We have also seen a similar phenotype when NICD was overexpressed. Furthermore, we have observed that if Awh is overexpressed along with Notch-ICD, Awh overexpression-induced cell death was also absent (no. of disc examined = 35; figure 9A1–C3). Awh transcript level was shown to be significantly downregulated in activated Notch condition (figure 1) [17]. Thus, we are inclined to speculate that feedback regulation between Awh and Notch may exist.

## 3. Discussion

The development of multicellular organisms relies on precisely regulated multiple cellular signal transduction pathways. The disruption in any signalling cascade may lead to catastrophic events for cells. To escape from such deleterious events, the cell has evolved multiple levels of intricate regulation of signalling pathways. Cross-talk between signalling pathways, signal integration and feedback mechanisms fine-tunes the outcome of signalling pathways.

Here we present a strong genetic interaction between *Awh* and *Notch* alleles. Awh loss-of-functions upregulates Notch signalling. Awh downregulates Notch signalling without altering the Notch receptor levels. It downregulates Notch signalling activity by downregulating the ligand, Delta. As a consequence of Notch signalling downregulation, Awh overexpression also downregulates Notch target Wg. Earlier it was also shown that there are increased transcripts of Awh in *Delta* mutant embryos, which supports the presence of a feedback loop between Notch and Awh [22]. Additionally, we found that the optimum amount of transcriptional co-factor Chip is critical for Awh function in wing patterning. Awh also rescues the neuronal anomalies caused by Notch gain-of-function. Furthermore, we observed that Awh activity gets compromised in activated Notch condition revealing a feedback mechanism between LIM-HD transcription factor Awh and Notch.

The Notch pathway has a profound role in different fundamental developmental events broadly classified as lateral inhibition, cell lineage decisions and boundary formation. Notch and Wg signalling pathways are crucial for DV boundary formation in both developing eye and wing imaginal discs. LIM-HD protein Apterous expression in the early wing primordium leads to expression of the Notch ligand Serrate in dorsal cells and restricts expression of another Notch ligand, Delta, to ventral cells [38]. Notch is symmetrically activated in cells on both sides of the DV compartment boundary by dorsally expressed Serrate and ventrally expressed Delta [38–40]. Expression of the glycosyltransferase, Fringe, makes dorsal cells more sensitive to Delta and less sensitive to Serrate [41,42]. Consequently, activated Notch induces Wg expression in cells along the DV boundary. Wg further activates expression of Serrate and Delta in nearby dorsal and ventral cells, respectively, and Serrate and Delta signal back to activate Notch and thereby maintain Cut and Wg expression along the DV boundary [43,44]. Thus, Notch and its ligands Delta and Serrate expression establishes a positive feedback loop that maintains signalling at the DV boundary in the wing imaginal discs. Similarly, a major role of Notch has been identified in boundary formation between the prospective somites during vertebrate somitogenesis [45] and at the midbrain–hindbrain boundary for organizer gene expression in chick embryos [46]. LIM-HD protein Awh along with transcriptional co-factor Chip together play a major role in restricting the eye field so that it helps in boundary formation between eye field and head surface at the ventral side [34]. Notch, Awh and Chip are very well conserved, and their orthologues are present from *Drosophila* to humans. Thus, we speculate that these proteins together fine-tune the boundary formation during metazoan development.

Notch was considered as a neurogenic gene since *Notch* mutants display a hyperplasia of the nervous system at expense of epidermal fate [47]. The orthologue of Awh, lhx6, has emerged as a probable candidate for Tourette syndrome, a neuro-muscular disease [48]. This prompted us to study the effect of Awh–Notch interplay on neuronal development. The neuronal defects of Notch gain-of-function were significantly reversed upon bringing Awh in the background. The climbing assay also supports a similar result. This observation opens up new avenues to study the Awh and Notch interaction in different neurodegenerative diseases in which dysregulation of the Notch pathway is involved.

The present study highlights the Awh and Notch feedback regulation and its functional implication in wing patterning, morphogenesis and neurogenesis.

## 4. Material and methods

### 4.1. *Drosophila* genetics

All fly stocks were maintained on standard cornmeal/yeast/molasses/agar medium at 25°C. *w^1118^* was used as wild-type controls. *UAS-Awh-RNAi* was a kind gift from Prof. L. S. Shashidhara. *C96GAL4::UAS-Mam(H), UAS-NICD, UAS-Notch DN* (dominant-negative Notch) and Notch pathway components, *N^nd-3^* and *N^54l9^,* were kindly provided by Prof. S. Artavanis-Tsakonas, Department of Cell Biology, Harvard Medical School. *UAS-Awh* was obtained as a gift from Prof. Ella Preger-Ben Noon, Howard Hughes Medical Institute., Ashburn, VA, USA. *Awh^63Ea-12^* was a kind gift from Prof. Hiroshi Shibuya, Simon Fraser University, Canada. *Dl^BX6^* was requested from Prof. Alexey Veraksa, University of Massachusetts Boston, USA. *UAS-HA-Chip* was previously generated in the lab [35]. GAL4 driver lines, *ap-GAL4/CyO, C96-GAL4, en-GAL4::UAS-GFP, ptc-GAL4::UAS-GFP, ptc-GAL4::UAS-GFP::GAL80ts, nub-GAL4, Gmr-GAL4, ey-GAL4::UAS-GFP, elav-GAL4, vg-GAL4, Awh^16^, Awh^63Ea-1^, UAS-Chip-∆LID* and *UAS-Chip-∆DD,* were procured from the Bloomington *Drosophila* stock centre. *vg-GAL4::UAS-GFP* was made with the help of appropriate genetic crosses. *nub-GAL4::UAS-GFP* was requested from the Cytogenetics Laboratory, Department of Zoology, BHU. All the crosses were performed at 25°C.

To generate Awh gain-of-function clones,virgin females of *hsp70-flp; Act FRT y FRT-GAL4* were crossed to *UAS-Awh* males. Heat shock was given at 37°C for 12 min at 24 h after egg laying, and the third instar larval discs were analysed for GFP-marked clones.

For studying overexpression of genes using temperature-sensitive GAL80 system, the desired crosses were allowed for egg laying at 18°C. When embryos matured into late first instar larvae, they were transferred to 24°C for 24 h and again moved to 18°C. The larvae and adult offspring were further used for studies.

### 4.2. Adult eye imaging and wing mounting

Flies were anaesthetized and placed on a bridged slide. The eye images were captured using Leica MZ10F microscope.

For wing mounting, adult wings were dissected from the hinge region of flies with the help of needle and scalpel and washed in isopropanol. The wings were then immediately mounted using wing mounting media. Wing images were taken using a brightfield Nikon Eclipse Ni microscope.

### 4.3. Embryo collection

Embryos were collected on a 2% agar plate supplemented with 0.2% propionic acid and yeast paste. Embryos were washed with distilled water, dechorionated in sodium hypochlorite and fixed for 1 h in 1:1 heptane and 4% paraformaldehyde solution. Embryos were then devitellinized by replacing heptane with methanol followed by vigorous shaking. Further washing was done in methanol. Devitellinized embryos were stored in methanol at −20° C. Embryonic stages were identified as described in Campos-Ortega & Hartenstein [49].

### 4.4. Immunocytochemistry and confocal microscopy

*Drosophila* third instar larval tissues were dissected in cold 1× phosphate buffer saline (PBS) and fixed in 4% paraformaldehyde for 20 min at room temperature. This was followed by four times washing of the discs using a washing solution (0.1% BSA in Tri-PBS), for 15 min each. Blocking was performed using a blocking solution (PBST with 8% normal goat serum) for 30 min to 1 h. Primary antibody was added, and tissues were incubated with it overnight at 4°C. Next day, the tissues were again washed with washing solution four times at room temperature for 20 min each. This was followed by blocking for 1 h at room temperature. Tissues were then incubated in a secondary antibody for 90 min. After washing the tissues with the washing solution four times and one wash in 1× PBS at room temperature, DAPI (1 µg ml^−1^) was added for 20 min to overnight in the dark. The tissues were finally dissected in 1× PBS and incubated in 1,4-diazabicyclo(2.2.2) for overnight. The next day, mounting of the discs was done, and tissues were observed under LSM 780 laser scanning confocal microscope Zeiss (Carl Zeiss). The images were processed in Adobe Photoshop 7. Mouse anti-Cut (1:100, 2B10), mouse anti-Wg (1:100, 4D4), mouse anti-Arm (1:100, N2 7A1), mouse anti-En (1:100, 4D9), mouse anti-Futsch (1:100, 22C10), mouse anti-Dl (1:100, C594.9B) and mouse anti-NICD (1:300, C17.9C6) were procured from Developmental Studies Hybridoma Bank. Guinea pig anti-Sens (1:100) was a kind gift from Prof. Hugo Bellen, Houston, USA, Department of Molecular and Human Genetics, Baylor College of Medicine. Rabbit anti-Dpp (1:100; kindly gifted by Prof. Matthew Gibson, Stowers Institute for Medical Research, Kansas City, MO, USA), rabbit anti-DCP-1 (1:100, 9578; procured from Cell Signaling Technology) and rabbit anti-HA (1:100, SAB5600116; procured from Sigma-Aldrich),Rabbit anti-Vg (1:100) were used as primary antibodies. Alexa Fluor 555-conjugated goat anti-mouse IgG (1:200), Alexa Fluor 488-conjugated goat anti-mouse IgG (1:200), Alexa Fluor 555-conjugated goat anti-rabbit IgG (1:200), Alexa Fluor 555-conjugated goat anti-rat IgG (1:200) and Alexa Fluor 488-conjugated goat anti-rabbit IgG (1:200; all from Molecular Probes) were used to detect primary antibodies.

### 4.5. RNA isolation and quantitation of transcripts

Total RNA was isolated from adult heads using TRIzol reagent following the manufacturer’s recommended protocol (Sigma-Aldrich). RNA (2 μg) was treated with DNAse using 1.0 µl 10× reaction buffer, 0.5 µl DNAse (New England Biolabs) and 0.5 μl RNAse inhibitor (1000 U ml^−1^), and volume was adjusted to 10 µl using DEPC MilliQ water. The reaction mixture was incubated at 37°C for 1 h. For single-stranded cDNA preparation, 10 µl of DNAse-treated RNA was mixed with 1 µl M-MuLV reverse transcriptase enzyme (New England Biolabs), 2 µl of 60 μM random primers, 2 µl of 10× M-MuLV buffer, 1 µl of 10 mM dNTP and 1 µl of RNase inhibitor, and nuclease-free water was used to make the total volume to 20 µl. The mixture was incubated at 25°C for 5 min followed by a 42°C incubation for 1 h. This cDNA was used as a template DNA for semiquantitative and RT quantitative (RT-qPCR). RT-qPCR was carried out as per the manufacturer’s protocol (Applied Biosystems). A total of 10 μl of the reaction volume included 5 μl 2× SYBR green, 0.25 μl of each forward and reverse primer and 1 μl cDNA. PCR was performed using an ABI 7500 instrument. Data were normalized to rps17 before calculating the relative fold change.

Primers used for the study are as follows:

mβ_RT_Fw 5′-ACCGCAAGGTGATGAAGC-3′

mβ_RT_Re 5′-CTTCATGTGCTCCACGGTC-3′

mδ_RT_Fw 5′-ATGGCCGTTCAGGGTCAG-3′

mδ_RT_Re 5′-CCATGGTGTCCACGATG-3′

mγ_RT_Fw 5′-GTCCGAGATGTCCAAGAC-3′

mγ_RT_Re 5′-GACTCCAAGGTGGCAACC-3′

m3_RT_Fw 5′-ATGGTCATGGAGATGTCC-3′

m3_RT_Re 5′-GCACTCCACCATCAGATC-3′

m5_RT_Fw 5′-ATGGCACCACAGAGCAAC-3′

m5_RT_Re 5′-TGTCCATTCGCAGGATGG-3′

m7_RT_Fw 5′-GGCCACCAAATACGAGATG-3′

m7_RT_Re 5′-CAT CGC CAG TCT GAG CAA-3′

m8_RT_Fw 5′-GGAATACACCACCAAGACC-3′

m8_RT_Re 5′-CGCTGACTCGAGCATCTC-3′

rps_RT_Fw 5′-AAGCGCATCTGCGAGGAG-3′

rps_RT_Re 5’- CCTCCTCCTGCAACTTGATG-3'

Dl_RT_FP 5′-CATCGTGCAGGTTCACAGTT-3′

Dl_RT_Re-5′-GTGGCCTGGTAGTGCTTTAG-3′

Awh_RT_Fw-5′-TTATCTGGAGACGGTCGAGG-3′,

Awh_RT Re-5′-AGATCCTGGCCATCCGGATT-3′

### 4.6. Climbing assay

A cohort of *n* > 15 age-matched adult female flies for each genotype was collected and transferred to a graduated cylinder with a height of 20 cm and a diameter of 2 cm. The cylinder was tapped to collect the flies at the bottom, and the movements of the flies were recorded for a duration of 10 s. These procedures were repeated at least 10 times.

### 4.7. Acridine orange staining

Acridine orange was used to detect apoptosis in developing *Drosophila* imaginal discs. Imaginal discs from third instar larvae of the F1 progeny from different crosses were dissected out and washed briefly in cold 1× PBS (pH 7.2). These discs were incubated in acridine orange (1 µg ml^–1^; Sigma) for 1 min, followed by two gentle washes in 1× PBS. Then the discs were mounted and observed immediately under a Nikon Eclipse 80i fluorescence microscope at a 488 nm emission filter.

### 4.8. Statistical analysis

Intensity profiling in the *Drosophila* imaginal discs and quantification of the adult wing area was done using ImageJ. In total, three to five imaginal discs were used for the quantification purpose in each case. Integrated density/area of the domain indicated the intensity of the staining in confocal images. The RGB plot profiles feature of ImageJ was also used to measure the intensity of Notch signalling components in different genetic combinations compared to the internal control.

RGB plot profiles feature from plugins section was then used to plot intensity graphs. The error bars in the graphs denoted the standard error of the mean value from the replicated experiments. Each dataset was repeated at least three times. *t*‐test and One-way analysis of variance with Tukey’s multiple comparison post-test were employed to determine the significance of the level of difference among the different genotypes. *p*‐value < 0.05 was accepted as statistically significant.

## Data Availability

The datasets supporting the conclusions of the article are included within the article. Supplementary material is available online [50].
